# Disability progression in multiple sclerosis: a latent class analysis of predictors

**DOI:** 10.1007/s00415-026-13704-5

**Published:** 2026-02-21

**Authors:** Jie Guo, Tomas Olsson, Lars Alfredsson, Anna Karin Hedström

**Affiliations:** 1 Department of Nutrition and Health, China Agricultural University, Beijing, China; 2 Department of Clinical Neuroscience, Karolinska Institutet, Stockholm, Sweden; 3 Institute of Environmental Medicine, Karolinska Institutet, Stockholm, Sweden; 4 Centre for Occupational and Environmental Medicine, Region Stockholm, Stockholm, Sweden

**Keywords:** Multiple sclerosis, Disability progression, Prognosis, Clinical and lifestyle predictors, Prospective cohort, Latent class analysis, EDSS, Progression, Trajectories

## Abstract

**Background:**

Multiple sclerosis (MS) is a heterogeneous disease with highly variable long-term outcomes. We aimed to identify patterns of disability progression and their determinants to improve individualized risk assessment and support clinical decision-making.

**Methods:**

We applied latent class trajectory modeling to Expanded Disability Status Scale (EDSS) data from 3163 newly diagnosed relapsing-onset MS cases in the Swedish Epidemiological Investigation of MS (2005–2019). Baseline demographics, clinical, and lifestyle data were collected at diagnosis, and participants were followed through the Swedish MS registry. Multivariate logistic regression was used to examine associations between baseline characteristics and trajectory group membership. Kaplan–Meier analysis estimated time to confirmed disability worsening, EDSS 3 and EDSS 4 across trajectory groups.

**Results:**

Seven distinct EDSS trajectories were identified. Group 4 (*n* = 1149) was the most common and characterized by stable, low disability. Groups 5–7 showed steadily increasing disability and were categorized as unfavorable (*n* = 662). In multivariate models, older age at diagnosis, longer baseline disease duration, higher baseline EDSS score, more frequent early relapses, poor cognitive performance, and autoimmune comorbidities were associated with unfavorable progression. Among lifestyle factors, obesity, smoking, and low sun exposure were linked to worse outcomes, while high physical activity decreased the odds of unfavorable progression. No significant association was observed for baseline vitamin D levels. Time to confirmed disability progression outcomes differed substantially across trajectory groups.

**Discussion:**

Early clinical and lifestyle factors were associated with long-term outcomes. These findings illustrate the heterogeneity of MS progression and support further investigation of early modifiable factors influencing long-term outcomes.

**Supplementary Information:**

The online version contains supplementary material available at 10.1007/s00415-026-13704-5.

## Introduction

Multiple sclerosis (MS) is a chronic, immune-mediated disease of the central nervous system, characterized by substantial heterogeneity in both clinical presentation and long-term outcomes [1]. While some individuals remain stable for extended period, others accumulate irreversible disability early in the disease course. Genetic variation appears to account for only a small share of these differences. Despite strong effects on susceptibility, the largest genome-wide study of MS severity identified only a single locus with modest impact on progression [2]. Accordingly, improving prognostic precision will require identifying determinants beyond genetics.

Predictive models have traditionally focused on baseline clinical characteristics such as older age at onset, male sex, early relapse activity, and initial disability [3–6]. More recently, modifiable lifestyle factors have emerged as relevant contributors to adverse outcomes [7–9]. In addition, accumulating evidence suggests that early initiation of disease-modifying treatment is associated with a reduced risk of long-term disability progression [10, 11]. However, most studies have modeled disease progression using time-to-event analyses or linear mixed-effects models, approaches that may not fully capture the heterogenous and dynamic nature of disability accumulation.

A trajectory-based approach offers a complementary perspective by identifying subgroups of individuals who follow similar patterns of disability evolution over time. This enables a more nuanced understanding of disease heterogeneity and may improve risk stratification beyond conventional prognostic markers. To date, only one study has applied this method to longitudinal Expanded Disability Status Scale (EDSS) data in individuals with relapsing–remitting MS (RRMS), identifying three distinct progression pathways over a 10-year period [12]. While that study assessed both clinical and lifestyle predictors of trajectory membership, associations with lifestyle factors were not observed, possibly due to limited sample size (*n* = 263). Whether similar patterns are present in other RRMS populations, and to what extent baseline clinical and lifestyle-related characteristics shape these trajectories, remains to be clarified.

In this study, we applied latent class trajectory modeling to longitudinal EDSS data from a population-based cohort of 3163 individuals with incident RRMS. Our aim was to identify distinct patterns of disability progression and examine their association with baseline clinical and lifestyle-related factors.

## Methods

We included participants from the Epidemiologic Investigation of Multiple Sclerosis (EIMS), a population-based case–control study conducted in Sweden. Individuals with incident MS were recruited between April 2005 and December 2019 from neurology departments across the country, including all university hospitals. A total of 3567 individuals with newly diagnosed MS were enrolled, with a response rate of 93% among cases. All MS diagnoses were confirmed by neurologists according to the McDonald criteria (2001 and later revisions) [13, 14]. At inclusion, participants completed a standardized questionnaire capturing information on lifestyle factors and environmental exposures. Blood samples were collected for genetic and serologic analysis. Detailed descriptions of the EIMS study design and data collection procedures have been published elsewhere1 [15].

For the present analysis, we excluded individuals with fewer than three recorded EDSS assessments within 15 years of diagnosis (*n* = 246) and those with a progressive-onset disease course (*n* = 158), resulting in a final sample of 3,163 individuals with relapsing-onset MS. The decision to exclude those with progressive-onset MS was made to ensure a clinically homogeneous sample and to avoid conflating distinct disease trajectories in the latent class modeling.

### Standard protocols approvals

The study was approved by the Regional Ethical Review Board at Karolinska Institute (reference number 2004–252/1–4 and 2013/1691–32) and has been conducted in accordance with the 1964 Declaration of Helsinki and subsequent revisions. All participants provided informed consent.

### Variables and covariates

Clinical data were retrieved from the Swedish MS register, a nationwide system used across all neurology departments in Sweden as part of routine clinical documentation [16]. The register includes prospectively recorded information on medical treatment, disease activity, and disability status, including repeated assessments of EDSS and Symbol Digit Modalities Test (SDMT). For this study, clinical variables included age at diagnosis, sex, disease duration (defined as the time from first symptoms to study baseline), baseline EDSS score, and the number of early relapses (within 5 years from disease onset), and baseline SDMT scores. Information on autoimmune comorbidities (rheumatoid arthritis, systemic lupus erythematosus, Sjögren’s syndrome, psoriasis, autoimmune thyroid disorders, diabetes mellitus type 1, ulcerous colitis, or Crohn’s disease) was collected through the EIMS questionnaire and was dichotomized into yes or no, and a history of infectious mononucleosis was defined based on participant recall of a prior clinical diagnosis. Treatment exposure was categorized according to the first disease-modifying therapy (DMT) initiated following diagnosis, grouped as untreated, platform therapy, or high-efficacy therapy. Platform therapy included interferon-beta, glatiramer acetate, teriflunomide, and dimethyl fumarate. High-efficacy DMTs included natalizumab, fingolimod, ocrelizumab, alemtuzumab, cladribine, mitoxantrone, and rituximab. Time from disease onset to treatment initiation was also included as a continuous variable.

Lifestyle-related variables included physical activity, body mass index, smoking, and sun exposure. Physical activity at diagnosis was self-reported on a four-level scale, with levels 3 and 4 indicating regular exercise that induces sweating. For analysis, responses were dichotomized into low (levels 1–2) and high (levels 3–4) physical activity. BMI was calculated by dividing self-reported weight in kilograms by height in meter squared and categorized into normal weight (BMI < 25), overweight (BMI 25–30), and obesity (BMI > 30 kg/m^2^). Smoking was dichotomized into baseline current smokers or non-smokers. Sun exposure was assessed using three questions regarding ultraviolet radiation exposure, with responses recorded on a four-point scale. The responses were summed up to create a composite index ranging from 3 to 12, representing overall sun exposure. The lowest sun exposure (3) was defined as low sun exposure.

Self-reported education level, validated by information retrieved from the National Board of Health and Welfare, was dichotomized at the threshold of obtaining a university degree. Education level at baseline and passive smoking exposure were included in the descriptive baseline table but were not tested as predictors. Education was previously found not to be independently associated with disease progression in this cohort after adjustment for lifestyle and treatment. Passive smoking was excluded due to its limited interpretability without stratifying or excluding active smokers.

For a subset of 974 patients recruited between 2005 and 2009, vitamin D status was assessed by measuring serum 25-hydroxyvitamin D levels using a chemiluminescent immunoassay (LIASON, Diasorin AB, Sundbyberg, Sweden), which quantifies both 25(OH)D₂ and 25(OH)D₃.

### Outcome measures

Long-term disability patterns were derived from repeated EDSS scores. Trajectory membership served as the primary outcome. We evaluated confirmed disability worsening (CDW), and time to first attainment of EDSS 3 and EDSS 4 across trajectory groups. CDW was defined as an EDSS increase from baseline sustained for at least 6 months (≥ 1.5 points if EDSS at baseline was 0, ≥ 1 point if baseline EDSS was 0.5–5.0, and ≥ 0.5 points if baseline EDSS was ≥ 5.5). Baseline EDSS was defined as the first EDSS within ± 6 months of diagnosis.

### Statistical analysis

We used latent class analysis to identify distinct groups of individuals with similar patterns of disability progression over time, based on repeated EDSS measurements. Time since diagnosis was used as the time scale. Models with increasing numbers of latent classes were estimated using maximum likelihood. Model fit was evaluated using a combination of Bayesian Information Criterion, clinical interpretability, entropy, and model parsimony. The final model included seven classes, each representing a unique trajectory of disability progression. Posterior probabilities were used to assign individuals to their most likely class. The quality of the classification was assessed using average posterior probability, odds of correct classification, and agreement between estimated and assigned class proportions [17, 18].

Baseline characteristics were summarized across trajectory groups using means and proportions. To simplify interpretation of long-term outcomes, we categorized the trajectory groups into favorable (groups 1–4) and unfavorable (groups 5–7) based on their overall disability level and trajectory slope. We then used binary logistic regression to identify baseline predictors of unfavorable progression. Covariates were selected based on theoretical relevance and statistical significance in univariable logistic regression models.

In the first multivariable model, we included core clinical variables: age at diagnosis, sex, disease duration, baseline EDSS score, number of early relapses, and autoimmune comorbidities. Treatment variables were not included at this stage, as they are not strictly baseline characteristics and may reflect early clinical decisions influenced by disease severity. In a second model, lifestyle variables were added. For simplicity, only variables that remained statistically significant were retained in the final multivariable model. A third model included additional adjustment for treatment (none, platform, or high-efficacy DMT) and time from disease onset to treatment initiation.

To explore the potential influence of cognitive performance and vitamin D status on long-term disability outcomes, we conducted two additional binary logistic regression models restricted to subsets of participants with relevant data. The first included individuals with available baseline SDMT scores (*n* = 2647), and the second included those with measured 25(OH)D concentrations at baseline (*n* = 974). Each model included the same set of clinical and lifestyle covariates as in the main analysis, along with the additional variable of interest. Because SDMT and vitamin D values were only available for a subset of the cohort, these variables were not included in the main multivariable model to avoid substantial loss of statistical power and potential selection bias.

As a supplementary analysis, we also performed a multinominal logistic regression using trajectory group 4 (the largest group with stable, low disability) as the reference category. Odds ratios (ORs) with 95% confidence intervals (CIs) were reported for each non-reference group.

To assess the clinical relevance of the trajectory classification, we conducted time-to-event analyses for CDW and for reaching EDSS 3 and EDSS 4 across trajectory groups. Hazards ratios (HRs) with 95% confidence intervals (CI) were reported. Follow-up time was calculated as the time from the baseline EDSS until the onset of the events of interest, drop-out, death, or end of follow-up (6 April 2022), whichever occurred first. The proportional hazard assumption was tested using Schoenfeld residuals, with no violations observed. Individuals who had already reached the respective EDSS threshold at baseline were excluded from each analysis. Survival functions were estimated using the Kaplan–Meier method, and median time to event with 95% confidence intervals was reported for each trajectory group, where calculable. Group differences were assessed using the log-rank test. All analyses were performed using SAS version 9.4 (SAS Institute, Cary, NC).

## Results

**Fig. 1:**
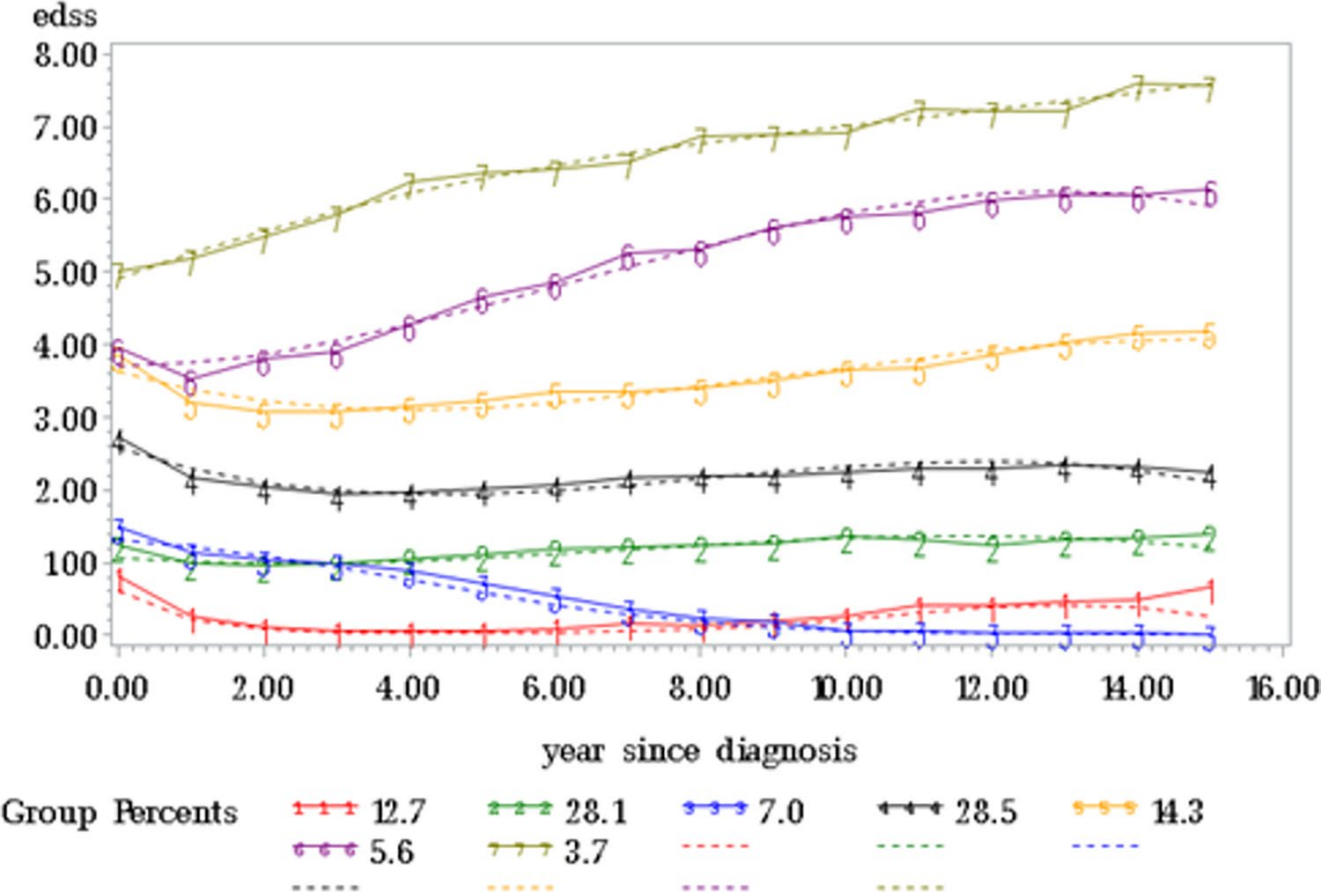
Distinct trajectories of disability progression (EDSS) over 15 years in patients with relapsing–remitting MS, identified using group-based trajectory modeling. Group-based trajectory modeling identified seven longitudinal patterns of disability progression based on the Expanded Disability Status Scale (EDSS). Each curve represents the estimated mean EDSS over time since diagnosis for one latent group. Group percentages indicate the proportion of the total cohort assigned to each trajectory. Solid lines represent observed group means, and dashed lines indicate model-estimated trajectories

Our analysis included 3163 individuals with newly diagnosed relapsing-onset MS. Participants were followed for a mean of 10.7 years (SD 3.8). The mean age at onset was 33.9 years, and 71.6% were women. Latent class trajectory modeling identified seven patterns of disability progression over time (Fig. 1). Model selection was based on Bayesian Information Criterion, clinical interpretability, entropy, and parsimony. Posterior probabilities for class assignment were acceptable across all groups (mean range 0.712–0.950) (eTable 1). Group 4 (*n* = 1149) represented the largest group and was characterized by low baseline disability and minimal or no progression during follow-up. Class-specific parameter estimates (intercept and slopes) for the seven trajectory groups are presented in eTable 2.

Baseline demographic, clinical, and lifestyle characteristics across the seven trajectory groups are presented in Table 1. Participants in the more severe trajectory groups were generally older at diagnosis, had higher EDSS scores at baseline, and more frequent early relapses. These groups also showed higher prevalence of smoking, higher BMI, lower physical activity, and lower sun exposure.

**Table 1:** Characteristics of total sample and by EDSS trajectory group

	Total	Group1	Group2	Group3
N	3163	368	830	154
Age at disease onset (SD)	37.1 (10.8)	33.4 (9.2)	34.5 (9.5)	32.0 (8.1)
Age at diagnosis (SD)	33.9 (10.2)	31.5 (8.6)	32.3 (9.5)	29.8 (7.8)
Baseline disease duration (SD)	3.1 (5.3)	1.8 (3.1)	2.1 (3.7)	2.2 (4.5)
Female, *n* (%)	2265 (71.6)	248 (67.4)	604 (72.7)	115 (74.7)
Nordic, *n* (%)	2524 (79.8)	303 (82.3)	643 (77.5)	125 (81.2)
University education, n (%)	1149 (36.3)	159 (43.2)	373 (44.9)	58 (37.7)
Autoimmune disorder, *n* (%)	329 (10.4)	21 (5.7)	77 (9.3)	18 (11.7)
Baseline EDSS (SD)	1.7 (1.4)	0.8 (1.0)	1.0 (0.9)	1.2 (1.0)
Mean number of relapses (SD)	2.0 (1.8)	1.4 (1.2)	2.0 (1.7)	2.3 (1.8)
Baseline MSIS-29 physical (SD)	19.8 (20.7)	6.8 (10.3)	10.7 (13.5)	9.7 (12.8)
Baseline MSIS-29 psychological (SD)	30.6 (23.9)	19.6 (20.0)	24.2 (20.9)	23.9 (21.4)
Baseline SDMT (SD)	52.4 (12.1)	57.4 (11.1)	54.8 (10.4)	55.1 (12.6)
Never treated, *n* (%)	91 (3.1)	8 (2.3)	15 (1.9)	4 (2.9)
Low-efficacy DMT, *n* (%)	2310 (78.1)	262 (74.6)	639 (81.3)	116 (82.7)
High-efficacy DMT, *n* (%)	556 (18.8)	81 (23.1)	132 (16.8)	20 (14.3)
Time between disease onset and DMT (SD)	3.3 (5.3)	1.8 (2.9)	2.4 (4.0)	2.2 (3.1)
Time between diagnosis and DMT (SD)	0.3 (1.5)	0.2 (0.9)	0.4 (1.6)	0.1 (1.0)
Proportion of follow-up on DMT (SD)	0.9 (0.2)	0.9 (0.2)	0.9 (0.2)	0.9 (0.2)
Past IM, *n* (%)	583 (18.5)	73 (20.0)	158 (19.0)	32 (20.9)
No past IM, *n* (%)	2205 (69.7)	262 (71.2)	578 (69.6)	105 (68.2)
Unknown IM history, *n* (%)	375 (11.9)	33 (9.0)	17 (11.3)	17 (11.0)
Non-smoker, *n* (%)	1486 (47.0)	209 (56.8)	412 (49.6)	78 (50.7)
Current smoker, *n* (%)	692 (21.9)	60 (16.3)	167 (20.1)	36 (23.4)
Past smoker, *n* (%)	985 (31.1)	99 (26.9)	251 (30.2)	40 (26.0)
Passive smoking, *n* (%)	1747 (55.2)	183 (49.7)	435 (52.4)	68 (44.2)
Normal weight; BMI < 25, *n* (%)	1859 (58.8)	220 (59.8)	526 (63.4)	96 (62.3)
Overweight; BMI 25–30, *n* (%)	859 (27.2)	103 (28.0)	216 (26.0)	42 (27.3)
Obesity; BMI > 30, *n* (%)	445 (14.1)	45 (12.3)	88 (10.6)	16 (10.4)
Physical activity score (SD)	2.4 (1.0)	2.7 (0.9)	2.5 (1.0)	2.6 (0.9)
Physical activity above median (SD)	1193 (37.7)	185 (50.3)	347 (41.8)	74 (48.1)
Sun exposure mean score (SD)	6.2 (1.8)	6.4 (1.8)	6.4 (1.7)	6.6 (1.8)
Low sun exposure (SD)	210 (6.6)	16 (4.4)	33 (4.0)	6 (3.9)
Baseline vitamin D (SD)	62.1 (26.9)	65.0 (25.8)	67.5 (28.4)	65.3 (23.5)

	Group4	Group5	Group6	Group7
N	1149	407	151	104
Age at disease onset (SD)	37.7 (11.0)	40.5 (11.2)	44.9 (9.8)	46.3 (10.5)
Age at diagnosis (SD)	34.3 (10.5)	36.5 (10.8)	39.2 (10.6)	38.9 (11.8)
Baseline disease duration (SD)	3.4 (5.6)	4.1 (6.3)	5.8 (6.6)	7.4 (8.8)
Female, *n* (%)	840 (73.1)	282 (69.3)	110 (72.9)	66 (63.5)
Nordic, *n* (%)	927 (80.7)	330 (81.1)	114 (75.5)	82 (78.9)
University education, *n* (%)	393 (34.2)	105 (25.8)	41 (27.2)	20 (19.2)
Autoimmune disorder, *n* (%)	119 (10.4)	54 (13.3)	20 (13.3)	20 (19.2)
Baseline EDSS (SD)	1.9 (1.2)	2.5 (1.6)	3.0 (1.3)	4.1 (1.7)
Mean number of relapses (SD)	1.8 (1.9)	2.6 (2.1)	2.6 (2.1)	1.8 (1.5)
Baseline MSIS-29 physical (SD)	21.8 (19.5)	32.2 (20.3)	45.8 (22.0)	54.0 (23.3)
Baseline MSIS-29 psychological (SD)	33.0 (23.4)	40.1 (24.3)	48.4 (25.0)	46.1 (27.6)
Baseline SDMT (SD)	52.2 (12.2)	47.6 (11.6)	42.7 (12.4)	42.6 (10.2)
Never treated, *n* (%)	40 (3.8)	11 (2.9)	9 (6.5)	4 (4.0)
Low-efficacy DMT, *n* (%)	793 (74.9)	314 (82.2)	115 (82.7)	71 (71.0)
High-efficacy DMT, *n* (%)	226 (21.3)	57 (14.9)	15 (10.8)	25 (25.0)
Time between disease onset and DMT (SD)	3.4 (5.7)	4.4 (6.3)	6.5 (6.9)	7.1 (8.1)
Time between diagnosis and DMT (SD)	0.3 (1.8)	0.3 (0.9)	0.4 (1.2)	0.3 (1.1)
Proportion of follow-up on DMT (SD)	0.9 (0.3)	0.9 (0.2)	0.8 (0.3)	0.8 (0.3)
Past IM, *n* (%)	216 (18.9)	64 (15.7)	28 (18.7)	12 (11.5)
No past IM, *n* (%)	801 (69.7)	279 (68.6)	107 (70.9)	73 (70.2)
Unknown IM history, *n* (%)	132 (11.5)	64 (15.7)	16 (10.6)	19 (18.3)
Non-smoker, *n* (%)	532 (46.3)	158 (38.8)	59 (39.1)	38 (36.5)
Current smoker, *n* (%)	247 (21.5)	115 (28.3)	41 (27.2)	26 (25.0)
Past smoker, *n* (%)	370 (32.2)	134 (32.9)	51 (33.8)	40 (38.5)
Passive smoking, n (%)	652 (56.7)	252 (61.9)	90 (59.6)	67 (64.4)
Normal weight; BMI < 25, *n* (%)	678 (59.0)	217 (53.3)	71 (47.0)	51 (49.0)
Overweight; BMI 25–30, *n* (%)	301 (26.2)	112 (27.5)	54 (35.8)	31 (29.8)
Obesity; BMI > 30, *n* (%)	170 (14.8)	78 (19.2)	26 (17.2)	22 (21.2)
Physical activity score (SD)	2.4 (1.0)	2.2 (0.9)	1.9 (0.8)	1.8 (0.8)
Physical activity above median (SD)	428 (37.3)	115 (28.3)	30 (19.9)	14 (13.5)
Sun exposure mean score (SD)	6.1 (1.8)	6.0 (1.9)	5.7 (1.7)	5.5 (2.0)
Low sun exposure (SD)	79 (6.9)	40 (9.8)	17 (11.3)	19 (18.3)
Baseline vitamin D (SD)	60.9 (27.0)	58.1 (25.2)	54.7 (24.7)	49.9 (22.4)

To simplify interpretation, we categorized trajectory groups 1–4 as favorable and groups 5–7 as unfavorable. In multivariable binary logistic regression, older age at diagnosis, longer disease duration, higher baseline EDSS, more frequent early relapses, and autoimmune comorbidities were associated with increased odds of belonging to an unfavorable trajectory group. Among lifestyle factors, obesity, low sun exposure, and smoking were associated with unfavorable progression, whereas high physical activity was associated with reduced risk of unfavorable trajectories (Table 2). Further adjustment for treatment exposure and time to treatment initiation yielded similar results (eTable 3).

**Table 2:** Logistic regression comparing baseline predictors of progressive versus stable or favorable EDSS trajectories (*n* = 3163)

	OR (95% CI)^1^	OR (95% CI)^2^	OR (95% CI)^3^
Age at diagnosis	1.06 (1.05–1.07)	1.05 (1.04–1.06)	1.05 (1.05–1.08)
Baseline disease duration	1.07 (1.06–1.09)	1.02 (1.00–1.04)	1.02 (1.01–1.05)
Male	1.16 (0.96–1.40)	1.03 (0.83–1.29)	1.00 (0.83–1.38)
Autoimmune comorbidity	1.60 (1.24–2.06)	1.49 (1.10–2.01)	1.40 (1.10–2.16)
Baseline EDSS	2.41 (2.22–2.62)	2.28 (2.09–2.48)	2.22 (2.66–3.33)
Number of relapses	1.13 (1.09–1.18)	1.21 (1.16–1.27)	1.21 (1.08–1.19)
High physical activity	0.45 (0.37–0.55)	–	0.65 (1.20–2.00)
Overweight Obesity	1.34 (1.10–1.63) 1.77 (1.40–2.25)	–	1.12 (0.88–1.42) 1.37 (1.03–1.82)
Current smoking	1.51 (1.24–1.83)	–	1.56 (1.23–1.97)
Low sun exposure	2.29 (1.71–3.08)	–	1.41 (1.00–2.03)

Progressive trajectories include groups 5–7; stable/favorable trajectories include groups 1–4

*EDSS* Expanded Disability Status Scale, *OR* odds ratio, *CI* confidence interval

^1^unadjusted; ^2^clinical variables were entered simultaneously in the model; ^3^clinical and lifestyle variables were entered simultaneously in the model

In sensitivity analyses restricted to participants with available baseline SDMT scores (*n* = 2647), lower cognitive performance was associated with unfavorable progression (Table 3). In contrast, baseline 25(OH)D levels were not significantly associated with trajectory group among the 974 participants with available vitamin D measurements (Table 4).

**Table 3:** Logistic regression comparing baseline predictors of progressive versus stable or favorable EDSS trajectories

	OR (95% CI)^1^	OR (95% CI)^2^
Age at diagnosis	1.06 (1.05–1.07)	1.04 (1.03–1.05)
Baseline disease duration	1.07 (1.05–1.09)	1.02 (0.99–1.04)
Male	1.21 (0.98–1.49)	0.97 (0.75–1.26)
Autoimmune comorbidity	1.69 (1.27–2.24)	1.39 (1.00–1.97)
Baseline EDSS	2.52 (2.29–2.78)	2.24 (2.03–2.48)
Number of relapses	1.16 (1.11–1.21)	1.21 (1.15–1.27)
High physical activity	0.44 (0.36–0.55)	0.65 (0.50–0.84)
Overweight Obesity	1.42 (1.14–1.78) 2.07 (1.59–2.68)	1.19 (0.91–1.56) 1.58 (1.14–2.17)
Current smoking	1.49 (1.20–1.86)	1.36 (1.04–1.77)
Low sun exposure	2.40 (1.72–3.35)	1.33 (0.91–1.89)
Baseline SDMT	0.944 (0.935–0.952)	0.965 (0.955–0.974)

Individuals with SDMT assessments (*n* = 2647). Progressive trajectories include groups 5–7; stable/favorable trajectories include groups 1–4

*EDSS* Expanded Disability Status Scale, *OR* odds ratio, *CI* confidence interval, *SDMT* Symbol Digit Modalities Test

^1^unadjusted; ^2^clinical and lifestyle variables were entered simultaneously in the model

**Table 4:** Logistic regression comparing baseline predictors of progressive versus stable or favorable EDSS trajectories

	OR (95% CI)^1^	OR (95% CI)^2^
Age at diagnosis	1.06 (1.05–1.08)	1.05 (1.03–1.07)
Baseline disease duration	1.06 (1.04–1.09)	1.05 (1.03–1.07)
Male	1.14 (0.85–1.54)	0.90 (0.61–1.33)
Autoimmune comorbidity	1.64 (1.26–2.14)	1.62 (1.00–1.62)
Baseline EDSS	2.43 (1.00–2.11)	2.41 (2.08–2.78)
Number of relapses	1.03 (0.97–1.08)	1.10 (1.04–1.17)
High physical activity	0.48 (0.35–0.65)	0.64 (0.44–0.93)
Overweight Obesity	1.03 (0.75–1.40) 1.62 (1.09–2.41)	0.94 (0.75–2.07) 1.34 (0.68–2.21)
Current smoking	1.44 (1.07–1.93)	1.50 (1.03–2.17)
Low sun exposure	2.31 (1.40–3.78)	1.14 (0.60–2.21)
Baseline vitamin D	0.988 (0.983–0.993)	0.996 (0.990–1.003)

Individuals with available vitamin D status (*n* = 974). Progressive trajectories include groups 5–7; stable/favorable trajectories include groups 1–4

*EDSS* Expanded Disability Status Scale, *OR *odds ratio, *CI *confidence interval, *SDMT *Symbol Digit Modalities Test

^1^unadjusted; ^2^clinical and lifestyle variables were entered simultaneously in the model

A supplemental multinomial logistic regression comparing each trajectory group to the reference group (group 4) is presented in eTable 4. The overall pattern of associations with clinical and lifestyle factors was consistent with the binary model, although estimates were imprecise, reflecting smaller sample sizes.

Kaplan–Meier analyses were used to estimate time to CDW, EDSS 3 and EDSS 4 across trajectory groups (eTable5). Only group 2 and groups 4–7 had sufficient numbers of events to estimate median time to CDW, which ranged from 11.51 (95% CI 9.73–13.17) years in group 2 to 1.83 (95% CI 1.38–2.57) years in group 7. For EDSS 3 and 4, median time was calculable in groups 4–7 and showed a gradient consistency with the severity of the respective trajectory.

## Discussion

In this large, population-based cohort of individuals with incident RRMS, we identified seven distinct trajectories of disability progression over a 15 year period using latent class trajectory modeling. While the majority followed favorable courses with minimal long-term progression, a substantial minority exhibited early and sustained disability accumulation. Our findings provide novel insights into the heterogeneity of MS progression and highlight the role of both clinical and modifiable lifestyle factors in shaping long-term outcomes.

By capturing longitudinal profiles, trajectory modeling offers a more nuanced understanding of disease heterogeneity than single-point or time-to-event approaches and may ultimately support more personalized risk stratification and management. While previous studies have applied latent class trajectory modeling in progressive MS populations (PPMS and SPMS) [19, 20], only one study has examined EDSS trajectories in a mixed cohort including both RRMS and PPMS, identifying three progression patterns over 10 years [11]. Our analysis, restricted to individuals with RRMS, revealed a greater granularity in progression patterns, which may reflect differences in sample size, follow-up duration, population characteristics, or model specification.

Clinical predictors of unfavorable trajectories in our cohort were largely consistent with established prognostic markers [2–4]. Older age at diagnosis, higher baseline EDSS scores, and frequent early relapses were strongly associated with worse long-term outcomes. These markers likely capture early disease burden and underlying aggressiveness, reinforcing the importance of early recognition and intervention. In contrast to some earlier studies [5], sex was not a consistent predictor across trajectory groups. Findings remained similar following adjustment for treatment exposure and time to treatment initiation, suggesting that the identified clinical predictors exert an effect on progression independent of early therapeutic decision-making.

Importantly, we also identified several modifiable lifestyle factors that were independently associated with disability trajectories. High physical activity was linked to favorable outcomes, while obesity, smoking, and low sun exposure were associated with increased odds of belonging to an unfavorable trajectory group. These findings support and extend prior observational studies demonstrating associations between lifestyle behaviors and MS progression but go further by anchoring these associations in the context of long-term disability trajectories. Although causality cannot be inferred with certainty from observational data, these results suggest that lifestyle modification may offer a complementary strategy for reducing disease burden alongside pharmacological treatment.

Our sensitivity analyses further underscore the relevance of baseline cognitive function. Lower SDMT scores were associated with unfavorable trajectories, consistent with previous research showing that cognitive impairment predicts more aggressive disease [21]. By contrast, baseline 25-hydroxy-vitamin D levels at baseline were not significantly associated with long-term trajectories in our cohort. While observational evidence has raised the possibility of a protective effect of vitamin D on disease activity [22–24], its prognostic value for long-term disability remains uncertain. Our cohort included individuals early in the disease course, during the presumed inflammatory phase when vitamin D would be expected to exert its greatest effect. The absence of an association in this context, despite adjustment for other prognostic factors, suggests that vitamin D may be more relevant as a short-term modulator of disease activity rather than a determinant of long-term progression. This interpretation is consistent with trial data failing to show sustained benefits of vitamin D supplementation on long-term outcomes [25, 26].

The role of DMTs in shaping long-term disability trajectories also warrants consideration. Although early initiation of high-efficacy therapy has been associated with improved long-term outcomes in previous studies, treatment exposure did not substantially alter the associations between baseline clinical and lifestyle factors and trajectory membership in our analyses. This likely reflects several factors related to clinical practice and outcome definition. In routine care, the choice of DMT is strongly influenced by early disease severity, which may limit the ability to disentangle treatment effects from underlying disease aggressiveness. In addition, our trajectory modeling captures long-term patterns of disability accumulation, which may be less sensitive to short- or medium-term treatment effects than conventional relapse- or event-based outcomes. Moreover, baseline characteristics appeared to exert a strong influence on long-term trajectories, potentially outweighing differences related to initial treatment choice. Taken together, these findings do not argue against the effectiveness of DMTs, but rather suggest that early disease course and patient-related factors play a major role in shaping long-term disability patterns, even in the context of contemporary treatment strategies.

Several limitations should be acknowledged. Although posterior probabilities for class assignment were high across groups, classification uncertainty may still have affected smaller trajectory groups. Lifestyle data were based on self-report and collected at a single time point, which may not fully capture changes over time or cumulative exposures. This also introduces the potential for recall bias, although data were collected close to the time of diagnosis. As in all observational studies, the non-randomized nature of exposure assessment limits causal interpretation. It is important to consider the potential for reverse causation, particularly regarding physical activity and sun exposure. Individuals in less favorable trajectory groups had higher baseline EDSS scores, which may have limited their mobility and outdoor engagement at study entry. Consequently, lower physical activity and sun exposure in these groups could partly reflect early functional impairment rather than pre-existing risk factors. Adjustment for baseline EDSS likely minimized this bias, although residual confounding due to early subclinical progression cannot be fully excluded. Treatment exposure was accounted for in supplementary analyses, but residual confounding by indication cannot be entirely ruled out. In addition, excluding individuals without repeated EDSS assessments may have introduced some degree of selection bias. Finally, our findings may not be generalizable to progressive-onset MS or to settings with substantially different genetic backgrounds, environmental exposures, or healthcare systems.

In conclusion, this study illustrates the heterogeneity of long-term disability progression in RRMS and points to both clinical and lifestyle determinants as shaping factors. These findings support the value of early prognostic assessment and indicate that lifestyle factors may offer additional opportunities to improve long-term outcomes.

## Supplementary Information

Below is the link to the electronic supplementary material.

Supplementary file1 (DOCX 25 KB)

## Data Availability

Anonymized data underlying this article will be shared on reasonable request from any qualified investigator that wants to analyze questions that are related to the published article.
