# Supplementary material for: Disability progression in multiple sclerosis: a latent class analysis of predictors
Source: J Neurol. 2026 Feb 21;273(2):157. doi: 10.1007/s00415-026-13704-5 (PMC12923449; doi:10.1007/s00415-026-13704-5)
Supplement: Supplementary file 1 — Supplementary file1 (DOCX 25 KB) [file 415_2026_13704_MOESM1_ESM.docx]

eTable 1. Model performance metrics for EDSS latent class trajectories.

| Group | N | Size | AvePP | OCC | BIC | AIC | Entropy |
| --- | --- | --- | --- | --- | --- | --- | --- |
| 1 | 368 | 11.6 | 0.919 | 86.48 | -30463 | -30359 | 0.779 |
| 2 | 830 | 26.2 | 0.865 |  |  |  |  |
| 3 | 154 | 4.9 | 0.868 |  |  |  |  |
| 4 | 1149 | 36.3 | 0.712 |  |  |  |  |
| 5 | 407 | 12.9 | 0.894 |  |  |  |  |
| 6 | 151 | 4.8 | 0.909 |  |  |  |  |
| 7 | 104 | 3.3 | 0.950 |  |  |  |  |

Size=proportion of total; avePP=average posterior probability; OCC=odds of correct classification; BIC=Bayesian Information Criterion; AIC=Akaike Information Criterion; Entropy=measure of classification certainty, ranging from 0 to 1 (higher values indicate better separation between classes).

eTable 2. Class-specific β coefficients and differences relative to the reference trajectory group

| Group | β coefficient | Difference in β coefficient | P value |
| --- | --- | --- | --- |
| 1 | -0.007 (-0.015, 0.001) | -0.041 (-0.051, -0.032) | <0.0001 |
| 2 | 0.029 (0.025, 0.033) | -0.006 (-0.012, 0.001) | 0.081 |
| 3 | -0.106 (-0.117, -0.096) | -0.141 (-0.152, -0.129) | <0.0001 |
| 4 | 0.035 (0.030, 0.039) | 1.0 (reference) |  |
| 5 | 0.086 (0.080, 0.093) | 0.052 (0.044, 0.060) | <0.0001 |
| 6 | 0.219 (0.208, 0.229) | 0.184 (0.173, 0.195) | <0.0001 |
| 7 | 0.215 (0.200, 0.230) | 0.181 (0.165, 0.196) | <0.0001 |

β coefficients represent the class-specific slope estimates from the latent class trajectory model. Differences in β coefficients were calculated relative to Group 4, the largest trajectory group. Values shown are estimates with 95% confidence intervals. P values correspond to Wald tests comparing class-specific slope estimates with the reference trajectory group (Group 4).

eTable 3. Logistic regression comparing baseline predictors of progressive versus stable or favorable EDSS trajectories. Individuals on DMT (n=3,072).

| Predictors | OR (95% CI)^1^ | OR (95% CI)^2^ |
| --- | --- | --- |
| Age at diagnosis | 1.06 (1.05-1.07) | 1.05 (1.03-1.06) |
| Baseline disease duration | 1.07 (1.06-1.09) | 0.99 (0.96-1.03) |
| Male, n (%) | 1.13 (0.93-1.38) | 0.99 (0.78-1.25) |
| Autoimmune comorbidity | 1.64 (1.26-2.14) | 1.34 (1.00-1.87) |
| Baseline EDSS | 2.47 (2.26-2.70) | 2.33 (2.13-2.55) |
| Number of relapses | 1.14 (1.09-1.18) | 1.19 (1.13-1.25) |
| High physical activity | 0.47 (0.38-0.57) | 0.65 (0.51-0.82) |
| Overweight  Obesity | 1.37 (1.12-1.69) 1.78 (1.38-2.30) | 1.09 (0.8851.39) 1.40 (1.04-1.88) |
| Current smoking | 1.51 (1.23-1.86) | 1.59 (1.25-2.02) |
| Low sun exposure | 2.45 (1.80-3.33) | 1.59 (1.09-2.33) |
| High-efficacy DMT | 0.77 (0.60-0.97) | 0.62 (0.46-0.84) |
| Time to treatment | 1.07 (1.06-1.09) | 1.04 (1.00-1.07) |

^1^unadjusted; ^2^clinical and lifestyle variables were entered simultaneously in the model; EDSS=expanded disability status scale; OR=odds ratio; CI=confidence interval; DMT=disease-modifying therapy; SDMT=Symbol Digit Modalities Test. Progressive trajectories include group 5-7; stable/favorable trajectories include groups 1-4.

eTable 4. Multinomial logistic regression comparing baseline predictors of disability trajectories (reference=group 4, n=1,149).

|  | Group1 | Group2 | Group3 |
| --- | --- | --- | --- |
|  | 368 | 830 | 154 |
| Age at diagnosis | 0.97 (0.95-0.98) | 0.98 (0.97-0.99) | 0.95 (0.93-0.97) |
| Baseline disease duration | 0.94 (0.90-0.97) | 0.95 (0.93-0.98) | 0.98 (0.94-1.03) |
| Male, n (%) | 0.74 (0.56-0.98) | 0.89 (0.72-1.11) | 0.97 (0.65-1.45) |
| Autoimmune | 0.58 (0.35-0.96) | 0.89 (0.65-1.23) | 1.28 (0.74-2.19) |
| Baseline EDSS | 0.35 (0.30-0.40) | 0.45 (0.41-0.50) | 0.58 (0.49-0.69) |
| Number of relapses | 0.85 (0.78-0.92) | 1.05 (0.99-1.09) | 1.10 (1.02-1.19) |
| High physical activity | 1.51 (1.17-1.97) | 1.09 (0.89-1.33) | 1.46 (1.03-2.07) |
| Overweight  Obesity | 1.12 (0.83-1.52) 0.93 (0.62-1.39) | 0.97 (0.77-1.22) 0.72 (0.53-0.98) | 1.19 (0.80-1.79) 0.85 (0.48-1.51) |
| Current smoker | 0.67 (0.48-0.94) | 0.88 (0.69-1.11) | 0.99 (0.66-1.49) |
| Low sun exposure | 0.70 (0.38-1.28) | 0.67 (0.43-1.03) | 0.72 (0.30-1.70) |

|  | Group5 | Group6 | Group7 |
| --- | --- | --- | --- |
|  | 407 | 151 | 104 |
| Age at diagnosis | 1.03 (1.02-1.04) | 1.06 (1.04-1.08) | 1.05 (1.03-1.07) |
| Baseline disease duration | 1.00 (0.98-1.02) | 1.02 (0.99-1.04) | 1.04 (1.01-1.07) |
| Male, n (%) | 0.89 (0.68-1.15) | 1.24 (0.82-1.87) | 0.90 (0.56-1.49) |
| Autoimmune | 1.29 (0.90-1.84) | 1.24 (0.72-2.11) | 1.89 (1.03-3.48) |
| Baseline EDSS | 1.53 (1.38-1.68) | 1.92 (1.67-2.20) | 2.78 (2.38-3.25) |
| Number of relapses | 1.21 (1.15-1.28) | 1.28 (1.19-1.38) | 1.12 (1.00-1.26) |
| High hysical activity | 0.79 (0.61-1.03) | 0.54 (0.35-0.84) | 0.40 (0.21-0.75) |
| Overweight  Obesity | 1.04 (0.78-1.38) 1.29 (0.93-1.79) | 1.43 (0.95-2.15) 1.19 (0.72-1.99) | 1.11 (0.65-1.90) 1.30 (0.71-2.39) |
| Baseline current smoker | 1.48 (1.13-1.94) | 1.50 (1.00-2.26) | 1.25 (0.74-2.14) |
| Low sun exposure | 1.20 (0.78-1.82) | 1.23 (0.67-2.24) | 1.72 (0.90-3.29) |

|  |  |  |  |
| --- | --- | --- | --- |

eTable 5. Time to CDW and EDSS milestones by EDSS trajectory group.

| Time to confirmed disability worsening | | | |
| --- | --- | --- | --- |
| Group | N | CDW (%) | Median time (95% CI) |
| 1 | 368 | 39 | - |
| 2 | 830 | 360 | 11.51 (9.73-13.17) |
| 3 | 154 | 41 | - |
| 4 | 1149 | 444 | 8.82 (7.69-9.75) |
| 5 | 407 | 296 | 3.95 (3.29-4.59) |
| 6 | 151 | 138 | 3.30 (2.68-3.72) |
| 7 | 104 | 94 | 1.83 (1.38-2.57) |
| Time to reaching EDSS 3 | | | |
| Group | N | EDSS 3 (%) | Median time (95% CI) |
| 1 | 348 | 1 | - |
| 2 | 805 | 45 | - |
| 3 | 142 | 10 | - |
| 4 | 929 | 368 | 9.04 (8.18-9.75) |
| 5 | 222 | 222 | 2.50 (2.09-2.88) |
| 6 | 61 | 61 | 2.47 (1.80-3.00) |
| 7 | 23 | 23 | 1.32 (0.96-1.53) |
| Time to reaching EDSS 4 | | | |
| Group | N | EDSS 4 (%) | Median time (95% CI) |
| 1 | 365 | 0 |  |
| 2 | 828 | 1 |  |
| 3 | 151 | 4 |  |
| 4 | 1089 | 93 | 12.9 (12.36-13.44) |
| 5 | 357 | 242 | 8.08 (7.11-8.49) |
| 6 | 112 | 112 | 4.75 (4.22-6.13) |
| 7 | 47 | 47 | 2.89 (2.06-3.29) |

CDW=confirmed disability worsening, defined as a sustained increase in EDSS; EDSS=expanded disability status scale. Median time to event is shown in years with 95% confidence intervals (CI). “-“ indicates that median time could not be estimated due to low event rates in the group.
